# The genome sequence of the grey wolf, *Canis lupus* Linnaeus 1758

**DOI:** 10.12688/wellcomeopenres.17332.1

**Published:** 2021-11-12

**Authors:** Mikkel-Holger S. Sinding, Shyam Gopalakrishnan, Katrine Raundrup, Love Dalén, Jonathan Threlfall, Darwin Tree of Life Barcoding collective, Wellcome Sanger Institute Tree of Life programme, Wellcome Sanger Institute Scientific Operations: DNA Pipelines collective, Tree of Life Core Informatics collective, Darwin Tree of Life Consortium, Tom Gilbert

**Affiliations:** 1 Globe Institute, University of Copenhagen, Copenhagen, Denmark; 2 Greenland Institute of Natural Resources, Kivioq 2, Nuuk, Greenland; 3 Center for Evolutionary Hologenomics, University of Copenhagen, Copenhagen, Denmark; 4 Centre for Palaeogenetics, Stockholm, Sweden; 5 Department of Bioinformatics and Genetics, Swedish Museum of Natural History, Stockholm, Sweden; 6 Department of Zoology, Stockholm University, Stockholm, Sweden; 7 Tree of Life, Wellcome Sanger Institute, Cambridge, UK; 8 Norwegian University of Science and Technology, Trondheim, Norway

**Keywords:** Canis lupus, Canis lupus orion, grey wolf, Polar wolf, Greenland wolf, genome sequence, chromosomal

## Abstract

We present a genome assembly from an individual male *Canis lupus orion *(the grey wolf, subspecies: Greenland wolf; Chordata; Mammalia; Carnivora; Canidae). The genome sequence is 2,447 megabases in span. The majority of the assembly (98.91%) is scaffolded into 40 chromosomal pseudomolecules, with the X and Y sex chromosomes assembled.

## Species taxonomy

Eukaryota; Metazoa; Chordata; Craniata; Vertebrata; Euteleostomi; Mammalia; Eutheria; Laurasiatheria; Carnivora; Caniformia; Canidae; Canis; *Canis lupus* Linnaeus 1758 (NCBI:txid9612).

## Background

The grey wolf, *Canis lupus*, is the largest species within the group wolf-like canids (Subtribe: *Canina*) and the member with the largest geographic distribution. Originally wolves were found throughout Eurasia, with the exception of tropical Southeast Asia, and all of North America. This vast distribution contains numerous habitats, encompassing wolf ecotypes adapted to the diverse environments throughout their distribution. The wolf is locally extinct in several places, such as the UK, Ireland and Brittany, yet it still holds much of its original distribution; the global population is estimated to be in the order of 200–250 thousand individuals ( Jhala *et al*., 2018).

Once numerous, wolves were eradicated from the islands of Great Britain in the 15th century and Ireland in the 18th century. There have been proposals to reintroduce populations of wolves to the Scottish Highlands to manage populations of red deer, which have a negative effect on biodiversity through overgrazing ( Nilsen *et al*., 2007). The Scottish Highlands are considered to be the only location in Great Britain that could support a healthy population of wolves; however, objections of livestock owners are likely to prevent their reintroduction in the near future ( Wilson, 2004). The reintroduction of wolves elsewhere has led not only to the reestablishment of this apex predator, but also to marked improvements in biodiversity in the ecosystem as a whole ( Ripple *et al*., 2014). Wolves reintroduced into the Yellowstone National Park, Wyoming, USA, in 1995 predated grazing animals such as wapiti ( *Cervus canadensis*) that preserved grasslands. The subsequent changes in prey behaviour led to trophic cascades that resulted in the reestablishment of tree species and an associated increase in populations of species that rely directly and indirectly on this habitat ( Ripple & Beschta, 2012).

Wolves have historically been found in Northwest, Northeast and East Greenland ( Dawes *et al*., 1986). Wolves were extirpated from East Greenland through hunting by 1939 and were absent from this area for the next 40 years ( Marquard-Petersen, 2012). In around 1979, a pair of wolves travelled from the north of the island and began a recolonisation of East Greenland, establishing a population of around 23 wolves ( Marquard-Petersen, 2011). A recent assessment found no trace of wolves for a number of years in East Greenland, while a population of up to 32 animals is still found in the northernmost parts of Greenland. Since the population in East Greenland was located entirely within the Northeast Greenland National Park, affording the wolves legal protection, it is unlikely that this extinction event was driven by hunting ( Marquard-Petersen, 2021).

Domestic dogs share a common ancestor with Eurasian wolves around 33,000 years ago ( Skoglund *et al*., 2015; Wang *et al*., 2016). In this regard, the Greenland wolf or Polar wolf reference genome described herein is highly relevant for dog and/or Eurasian wolf genomics. The Polar wolf is a North American wolf, an outgroup to dogs and Eurasian wolves ( Gopalakrishnan *et al*., 2019; Sinding *et al*., 2018), which will aid in making a minimally reference-biased representation of diversity in re-sequenced genomes ( Gopalakrishnan *et al*., 2017). The Polar wolf is also the North American wolf type with the least coyote-like ancestry ( Sinding *et al*., 2018); thus, it is probably the closest possible outgroup to dogs and Eurasian wolves with the least amount of exotic admixture that other North American wolves carry. Finally, this reference genome permits detailed genomic investigations of Polar wolves themselves, as a precise reference, to identify rare genomic variation. The genome is therefore an overall useful resource for research in the Polar wolf itself, a small, isolated and understudied population, but also canids, wolves and dogs overall.

## Genome sequence report

The genome was sequenced from a single male *C. lupus* subspecies *orion* collected from Siorapaluk, Greenland (latitude 77.785278, longitude -70.631389) in 2016. A total of 28-fold coverage in Pacific Biosciences single-molecule long reads and 74-fold coverage in 10X Genomics read clouds were generated. Primary assembly contigs were scaffolded with chromosome conformation Hi-C data. Manual assembly curation corrected 135 missing/misjoins and removed 9 haplotypic duplications, reducing the assembly length by 0.2% and the scaffold number by 42.1%, and increasing the scaffold N50 by 15.9%.

**Figure 1.**
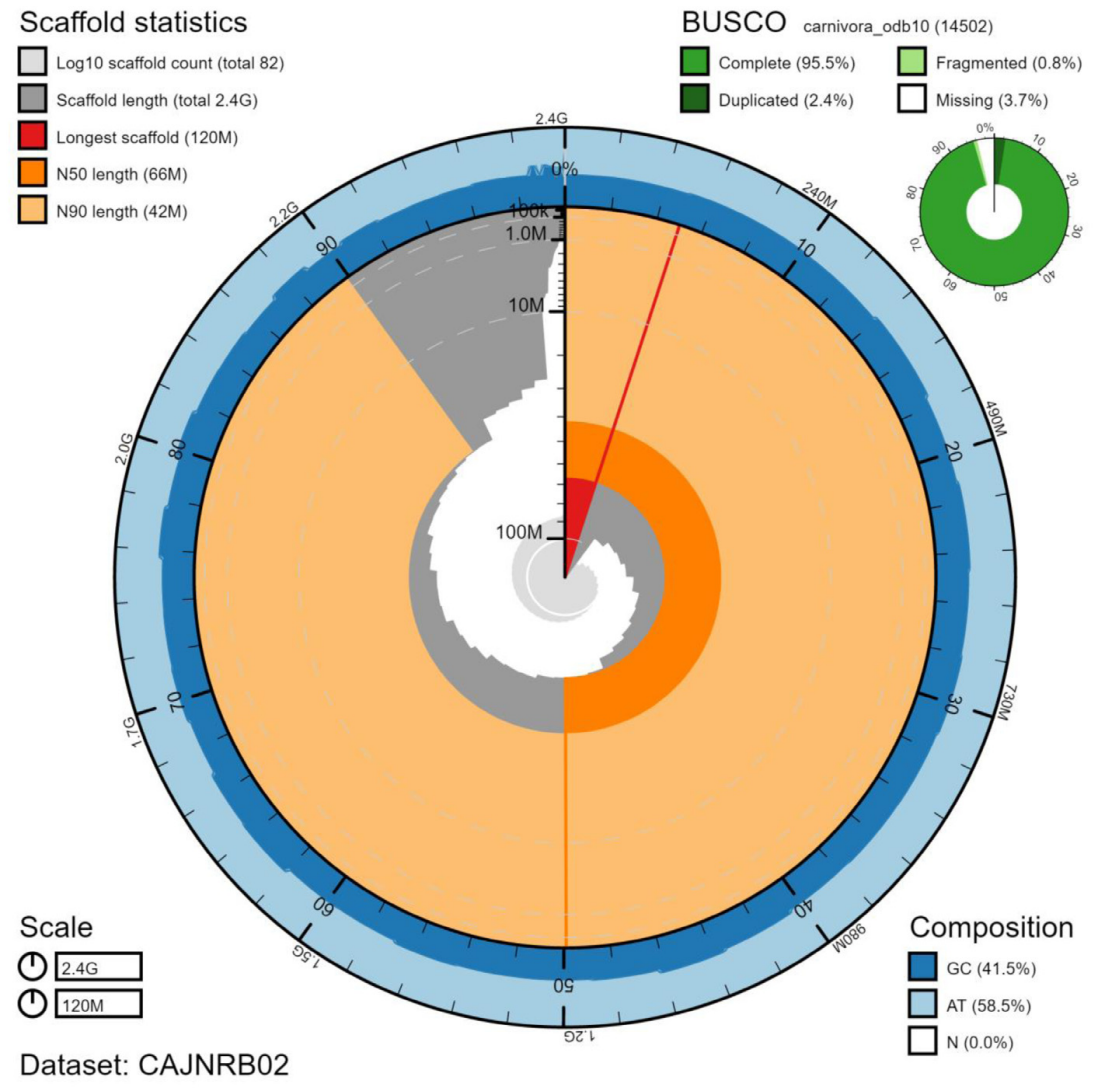
Genome assembly of *Canis lupus*, mCanLor1.2: metrics. The BlobToolKit Snailplot shows N50 metrics and BUSCO gene completeness. The main plot is divided into 1,000 size-ordered bins around the circumference with each bin representing 0.1% of the 2,447,463,909 bp assembly. The distribution of scaffold lengths is shown in dark grey with the plot radius scaled to the longest scaffold present in the assembly (124,665,963 bp, shown in red). Orange and pale-orange arcs show the N50 and N90 scaffold lengths (65,778,685 and 41,774,919 bp), respectively. The pale grey spiral shows the cumulative scaffold count on a log scale with white scale lines showing successive orders of magnitude. The blue and pale-blue area around the outside of the plot shows the distribution of GC, AT and N percentages in the same bins as the inner plot. A summary of complete, fragmented, duplicated and missing BUSCO genes in the carnivora_odb10 set is shown in the top right. An interactive version of this figure is available at https://blobtoolkit.genomehubs.org/view/mCanLor1.2/dataset/CAJNRB02/snail.

The final assembly has a total length of 2,447 Mb in 82 sequence scaffolds with a scaffold N50 of 66 Mb ( Table 1). Of the assembly sequence, 98.91% was assigned to 40 chromosomal-level scaffolds (named by synteny to an assembly for *C. lupus familiaris*, breed labrador: GCF_014441545.1), including 38 autosomes and the X and Y chromosomes ( Figure 1– Figure 4; Table 2). The assembly has a BUSCO ( Simão *et al*., 2015) completeness of 95.5% (single 93.0%, duplicated 2.4%) using the carnivora_odb10 reference set. While not fully phased, the assembly deposited is of one haplotype. Contigs corresponding to the second haplotype have also been deposited.

**Table 1.** Genome data for *Canis lupus*, mCanLor1.2.

*Project accession data*	
Assembly identifier	mCanLor1.2
Species	*Canis lupus*
Specimen	mCanLor1
NCBI taxonomy ID	NCBI:txid9612
BioProject	PRJEB43200
BioSample ID	SAMEA7532739
Isolate information	Male, muscle
*Raw data accessions*	
PacificBiosciences SEQUEL II	ERR6406204, ERR6406205, ERR6412029, ERR6412030, ERR6412359, ERR6412360
10X Genomics Illumina	ERR6054484-ERR6054491
Hi-C Illumina	ERR6511153
Illumina RNA-Seq	ERR6054492
*Genome assembly*	
Assembly accession	GCA_905319855.2
Accession of alternate haplotype	GCA_905319845.1
Span (Mb)	2,447
Number of contigs	248
Contig N50 length (Mb)	34
Number of scaffolds	82
Scaffold N50 length (Mb)	66
Longest scaffold (Mb)	123
BUSCO * genome score	C:95.8%[S:94.6%,D:1.2%], F:2.0%,M:2.2%,n:4104

*BUSCO scores based on the carnivora_odb10 BUSCO set using v5.1.2. C= complete [S= single copy, D=duplicated], F=fragmented, M=missing, n=number of orthologues in comparison. A full set of BUSCO scores is available at https://blobtoolkit.genomehubs.org/view/Canis%20lupus/dataset/CAJNRB02/busco.

**Figure 2.**
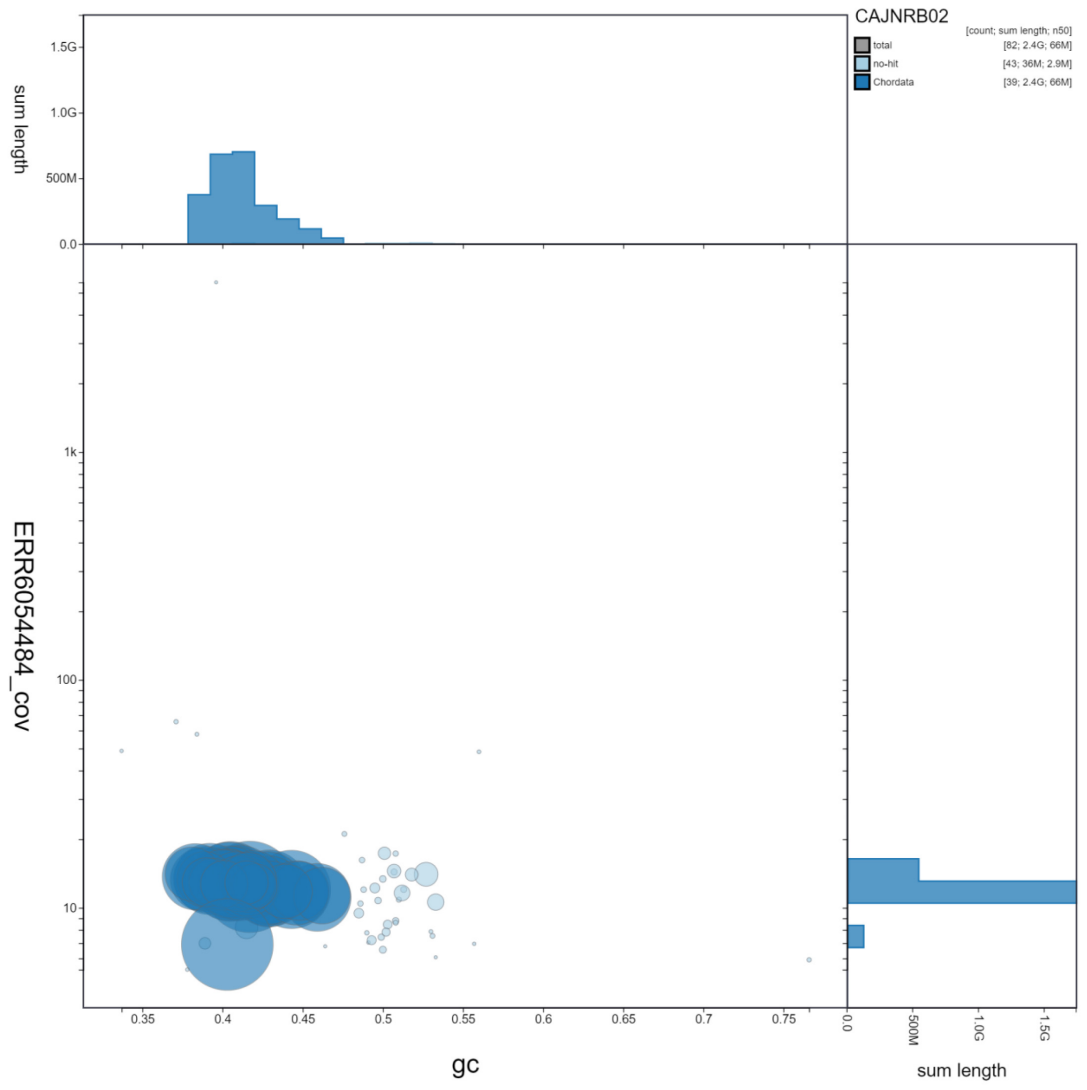
Genome assembly of *Canis lupus*, mCanLor1.2: GC coverage. BlobToolKit GC-coverage plot. Scaffolds are coloured by phylum. Circles are sized in proportion to scaffold length. Histograms show the distribution of scaffold length sum along each axis. An interactive version of this figure is available at https://blobtoolkit.genomehubs.org/view/mCanLor1.2/dataset/CAJNRB02/blob.

**Figure 3.**
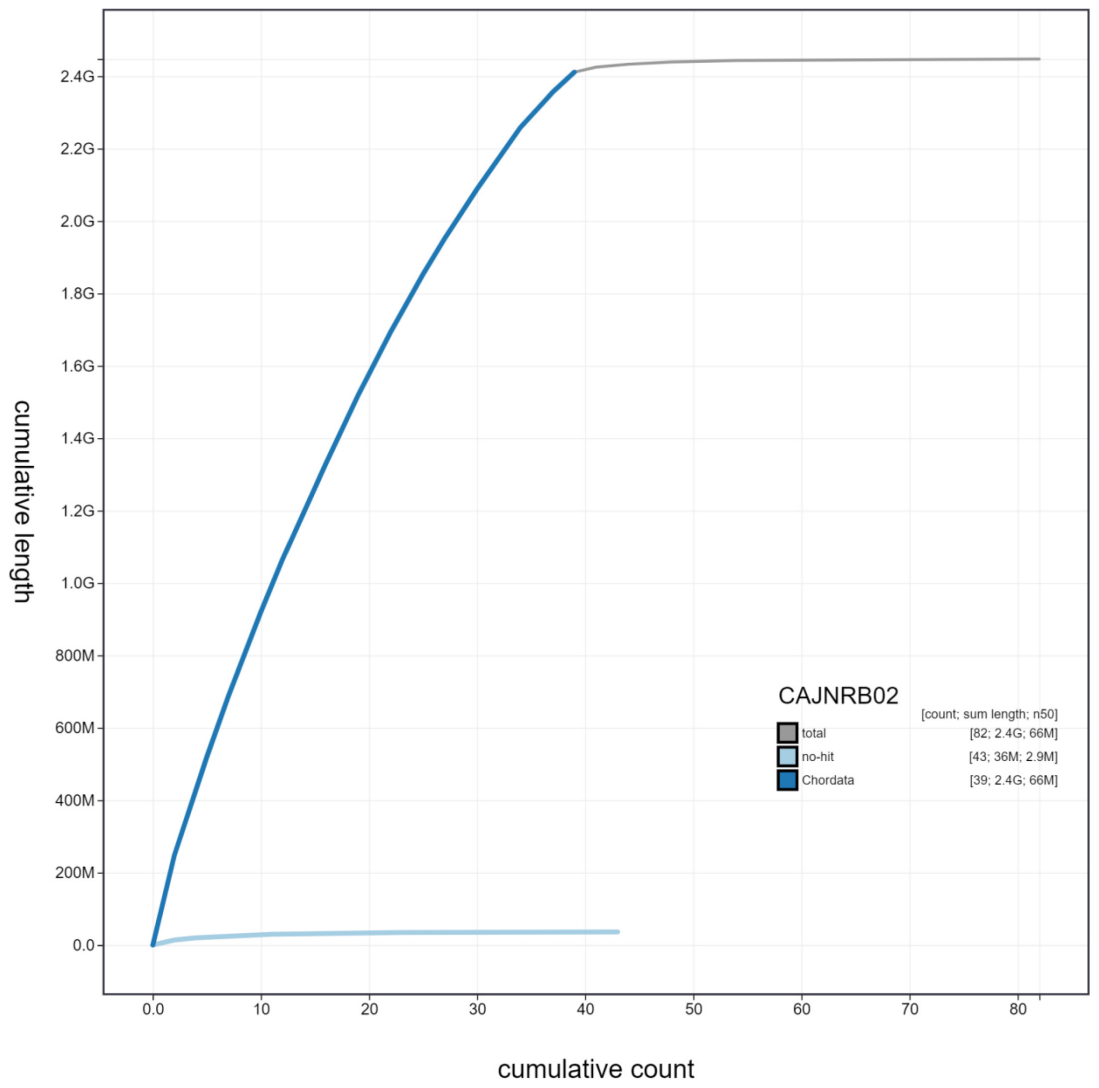
Genome assembly of *Canis lupus*, mCanLor1.2: cumulative sequence. BlobToolKit cumulative sequence plot. The grey line shows cumulative length for all scaffolds. Coloured lines show cumulative lengths of scaffolds assigned to each phylum using the buscogenes taxrule. An interactive version of this figure is available at https://blobtoolkit.genomehubs.org/view/mCanLor1.2/dataset/CAJNRB02/cumulative.

**Figure 4.**
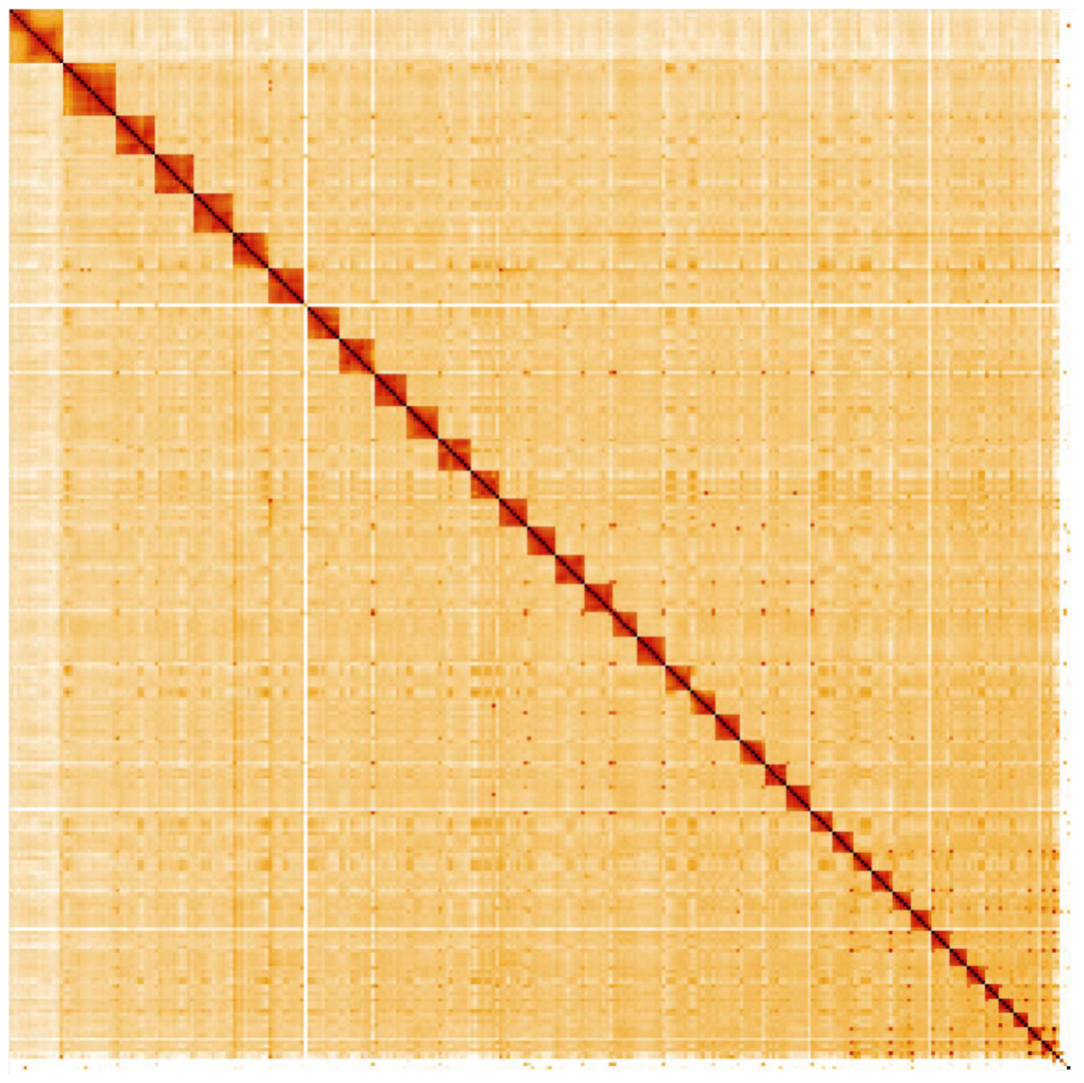
Genome assembly of *Canis lupus*, mCanLor1.2: Hi-C contact map. Hi-C contact map of the mCanLor1.2 assembly, visualised in HiGlass. Chromosomes are shown in size order from left to right and top to bottom.

**Table 2.** Chromosomal pseudomolecules in the genome assembly of *Canis lupus*, mCanLor1.2.

INSDC accession	Chromosome	Size (Mb)	GC%
HG994383.1	1	122.96	41.7
HG994387.1	2	86.40	42.9
HG994384.1	3	93.48	40.5
HG994386.1	4	88.63	40.4
HG994385.1	5	89.78	44.3
HG994389.1	6	78.39	42.8
HG994388.1	7	82.29	41.1
HG994390.1	8	77.59	40.8
HG994394.1	9	66.79	45.9
HG994393.1	10	71.93	42.9
HG994391.1	11	75.75	40.4
HG994392.1	12	73.73	39.2
HG994397.1	13	65.44	40.3
HG994400.1	14	62.79	39
HG994395.1	15	65.78	40.5
HG994398.1	16	63.67	41.5
HG994396.1	17	65.96	41.9
HG994402.1	18	57.59	43
HG994403.1	19	56.75	38.7
HG994401.1	20	59.77	44.6
HG994405.1	21	53.11	40.4
HG994399.1	22	63.45	38.3
HG994406.1	23	52.96	40
HG994407.1	24	49.88	44.7
HG994404.1	25	53.62	41.6
HG994409.1	26	46.11	46.2
HG994408.1	27	48.75	40.8
HG994413.1	28	42.48	43.9
HG994412.1	29	44.09	38.9
HG994415.1	30	41.62	41.6
HG994411.1	31	44.76	41
HG994414.1	32	41.77	38.1
HG994417.1	33	32.66	39.4
HG994410.1	34	45.90	41.6
HG994419.1	35	28.53	42
HG994416.1	36	33.43	39
HG994418.1	37	31.50	40.1
HG994420.1	38	26.44	41.5
HG994381.1	X	124.67	40.3
HG994382.1	Y	6.54	41.5
HG998573.1	MT	0.02	39.6
-	Unplaced	29.74	50.3

## Methods

A single 4-year-old male *C. lupus orion* (mCanLor1) was collected from Siorapaluk, Greenland (latitude 77.785278, longitude -70.631389) by The Ministry of Fisheries, Hunting and Agriculture, Government of Greenland. The animal was put down by the local municipal bailiff in Siorapaluk on 13 January 2016. The wolf had little fear of humans, persistently entered the village and could not be chased away. It was therefore decided that the wolf should be killed to protect villagers and dogs in Siorapaluk. After termination, the skull of the specimen was confiscated by the authorities and made available for the purposes of research to the Greenland Institute of Natural Resources.

DNA was extracted from the muscle tissue of mCanLor1 at the Wellcome Sanger Institute (WSI) Scientific Operations core from the whole organism using the Qiagen MagAttract HMW DNA kit, according to the manufacturer’s instructions. RNA (from the same muscle tissue) was extracted in the Tree of Life Laboratory at the WSI using TRIzol, according to the manufacturer’s instructions. RNA was then eluted in 50 μl RNAse-free water and its concentration RNA assessed using a Nanodrop spectrophotometer and Qubit Fluorometer using the Qubit RNA Broad-Range (BR) Assay kit. Analysis of the integrity of the RNA was done using Agilent RNA 6000 Pico Kit and Eukaryotic Total RNA assay.

Pacific Biosciences HiFi circular consensus and 10X Genomics read cloud sequencing libraries were constructed according to the manufacturers’ instructions. Poly(A) RNA-Seq libraries were constructed using the NEB Ultra II RNA Library Prep kit. DNA sequencing was performed by the Scientific Operations core at the Wellcome Sanger Institute on Pacific Biosciences SEQUEL II and Illumina HiSeq X instruments. RNA sequencing was performed using an Illumina MiSeq instrument. Further 10X sequencing was performed at SciLifeLab, Stockholm, Sweden. DNA was extracted using the automatic KingFisher™ Duo Prime Purification System (Thermo Fisher Scientific, Bremen, Germany) following the manufacturer's protocol. Following this, Illumina TruSeq PCR-free libraries were constructed and sequencing performed on HiSeq X. Hi-C data were generated at SciLifeLab, Stockholm, Sweden using the Dovetail Hi-C kit and sequenced on HiSeq X.

Assembly was carried out with Hifiasm ( Cheng *et al*., 2021). Haplotypic duplication was identified and removed with purge_dups ( Guan *et al*., 2020). Scaffolding with Hi-C data ( Rao *et al*., 2014) was carried out with SALSA2 ( Ghurye *et al*., 2019). The Hi-C scaffolded assembly was polished with the 10X Genomics Illumina data by aligning to the assembly with longranger align, calling variants with freebayes ( Garrison & Marth, 2012). One round of the Illumina polishing was applied. The mitochondrial genome was assembled with MitoHiFi ( Uliano-Silva *et al*., 2021). The assembly was checked for contamination and corrected using the gEVAL system ( Chow *et al*., 2016) as described previously ( Howe *et al*., 2021). Manual curation ( Howe *et al*., 2021) was performed using gEVAL, HiGlass ( Kerpedjiev *et al*., 2018) and Pretext. Regions of concern were identified and resolved using 10X longranger and genetic mapping data. The genome was analysed within the BlobToolKit environment ( Challis *et al*., 2020). Table 3 contains a list of all software tool versions used, where appropriate.

**Table 3.** Software tools used.

Software tool	Version	Source
Hifiasm	0.12	Cheng *et al*., 2021
purge_dups	1.2.3	Guan *et al*., 2020
SALSA2	2.2	Ghurye *et al*., 2019
longranger align	2.2.2	https://support.10xgenomics.com/ genome-exome/software/pipelines/latest/ advanced/other-pipelines
freebayes	1.3.1-17-gaa2ace8	Garrison & Marth, 2012
MitoHiFi	1	Uliano-Silva *et al*., 2021
gEVAL	N/A	Chow *et al*., 2016
PretextView	0.1.x	https://github.com/wtsi-hpag/PretextView
HiGlass	1.11.6	Kerpedjiev *et al*., 2018
BlobToolKit	2.6.2	Challis *et al*., 2020

## Data availability

European Nucleotide Archive: Canis lupus (Greenland wolf). Accession number PRJEB43200; https://identifiers.org/ena.embl/PRJEB43200.

The genome sequence is released openly for reuse. The *C. lupus* genome sequencing initiative is part of the Darwin Tree of Life (DToL) project and the Vertebrate Genomes Project. All raw sequence data and the assembly have been deposited in INSDC databases. The genome will be annotated using the RNA-Seq data and presented through the Ensembl pipeline at the European Bioinformatics Institute. Raw data and assembly accession identifiers are reported in Table 1.

## Author information

Members of the Darwin Tree of Life Barcoding collective are listed here: https://doi.org/10.5281/zenodo.4893704.

Members of the Wellcome Sanger Institute Tree of Life programme are listed here: https://doi.org/10.5281/zenodo.5377053.

Members of Wellcome Sanger Institute Scientific Operations: DNA Pipelines collective are listed here: https://doi.org/10.5281/zenodo.4790456.

Members of the Tree of Life Core Informatics collective are listed here: https://doi.org/10.5281/zenodo.5013542.

Members of the Darwin Tree of Life Consortium are listed here: https://doi.org/10.5281/zenodo.4783559.
