# Supplementary material for: The genome sequence of the grey wolf, Canis lupus Linnaeus 1758
Source: Wellcome Open Res. 2021 Nov 12;6:310. [Version 1] doi: 10.12688/wellcomeopenres.17332.1 (PMC8649967; doi:10.12688/wellcomeopenres.17332.1)
Supplement: Peer review history

# Peer review: Reviewer response for version 1

**DOI:** 10.21956/wellcomeopenres.19162.r47083

**Published:** 2021-12-06

**Authors:** Rémi Allio

**Affiliations:** 1 Montpellier Institute of Evolutionary Sciences (ISEM), CNRS, IRD, EPHE, University of Montpellier, Montpellier, France

The Data Note "The genome sequence of the grey wolf, *Canis lupus *Linnaeus 1758" presented by Sinding *et al. *is the description of the genome of the grey wolf obtained following the Darwin Tree of Life protocols. PacBio single-molecule long reads, 10X Genomics read clouds, Illumina reads and Hi-C data were generated and used to assemble this genome. RNA-seq libraries were also constructed and sequenced. However, the authors do not elaborate on why and where these are used (for Ensembl pipeline?).

 The resulting assembly is of high quality with the majority of the assembly (~99%) scaffolded into 40 chromosomal pseudomolecules. 

 However, given the genome provided here, I think a comparison (based on BUCSO scores or traditional measures such as number of scaffolds or N50) with other genomes available for the species *Canis lupus* would have been interesting. Indeed, even if this genome is the first available assembly for the subspecies *Canis lupus orion*, there are 25 other assemblies for this species available on NCBI, with among them, other chromosome length genome assemblies.

 Minor but mandatory comments:

- Explain the use of RNA-seq data. Is it only for use in the Ensembl pipeline?
- Not all softwares are presented in Table 3. At least BUSCO and its version are missing. 

Are sufficient details of methods and materials provided to allow replication by others?

Yes

Is the rationale for creating the dataset(s) clearly described?

Yes

Are the datasets clearly presented in a useable and accessible format?

Yes

Are the protocols appropriate and is the work technically sound?

Yes

Reviewer Expertise:

Phylogenomics and Genomics

I confirm that I have read this submission and believe that I have an appropriate level of expertise to confirm that it is of an acceptable scientific standard.

# Peer review: Reviewer response for version 1

**DOI:** 10.21956/wellcomeopenres.19162.r47087

**Published:** 2021-11-25

**Authors:** Michael Hiller

**Affiliations:** 1 LOEWE Centre for Translational Biodiversity Genomics, Frankfurt am Main, Germany; 2 Senckenberg Society for Nature Research, Frankfurt am Main, Germany; 3 Goethe University, Frankfurt am Main, Germany

The publication describes a high-quality genome of the Polar wolf, which will be an asset for population genomics, pangenomics of Canids and likely research aiming at understanding dog domestication. The study is well motivated, the methods are clearly described and the assembly is done by an expert team of genome scientists.

 I have only two suggestions:

1. The method section mentions that HiFi circular consensus reads were produced. Maybe this can be mentioned above in the genome report:
  "A total of 28-fold coverage in Pacific Biosciences single-molecule, circular consensus (HiFi) long reads ..."
2. Since 10X data was also produced and used for polishing, it makes sense to use Merqury to compute a base QV value. Maybe this can be added.

Are sufficient details of methods and materials provided to allow replication by others?

Yes

Is the rationale for creating the dataset(s) clearly described?

Yes

Are the datasets clearly presented in a useable and accessible format?

Yes

Are the protocols appropriate and is the work technically sound?

Yes

Reviewer Expertise:

genomics

I confirm that I have read this submission and believe that I have an appropriate level of expertise to confirm that it is of an acceptable scientific standard.
